# A cost-effective approach to produce ^15^N-labelled amino acids employing *Chlamydomonas reinhardtii CC503*

**DOI:** 10.1186/s12934-017-0759-9

**Published:** 2017-08-18

**Authors:** Jesús Nicolás Carcelén, Juan Manuel Marchante-Gayón, Pablo Rodríguez González, Luis Valledor, María Jesús Cañal, José Ignacio García Alonso

**Affiliations:** 1 Department of Physical and Analytical Chemistry, Faculty of Chemistry, University of Oviedo, Julián Clavería 8, 33006 Oviedo, Spain; 2 Department of Organisms and Systems Biology, University of Oviedo, C/Catedrático Rodrigo Uría s/n, 33071 Oviedo, Spain

**Keywords:** Biosynthesis, *Chlamydomonas reinhardtii*, ^15^N-labelled amino acids, Isotopic enrichment, Protein extraction, GC–MS

## Abstract

**Background:**

The use of enriched stable isotopes is of outstanding importance in chemical metrology as it allows the application of isotope dilution mass spectrometry (IDMS). Primary methods based on IDMS ensure the quality of the analytical measurements and traceability of the results to the international system of units. However, the synthesis of isotopically labelled molecules from enriched stable isotopes is an expensive and a difficult task. Either chemical and biochemical methods to produce labelled molecules have been proposed, but so far, few cost-effective methods have been described.

**Results:**

The aim of this study was to use the microalgae *Chlamydomonas reinhardtii* to produce, at laboratory scale, ^15^N-labelled amino acids with a high isotopic enrichment. To do that, a culture media containing ^15^NH_4_Cl was used. No kinetic isotope effect (KIE) was observed. The labelled proteins biosynthesized by the microorganism were extracted from the biomass and the ^15^N-labelled amino acids were obtained after a protein hydrolysis with HCl. The use of the wall deficient strain CC503 cw92 mt+ is fit for purpose, as it only assimilates ammonia as nitrogen source, avoiding isotope contamination with nitrogen from the atmosphere or the reagents used in the culture medium, and enhancing the protein extraction efficiency compared to cell-walled wild type *Chlamydomonas*. The isotopic enrichment of the labelled amino acids was calculated from their isotopic composition measured by gas chromatography mass spectrometry (GC–MS). The average isotopic enrichment for the 16 amino acids characterized was 99.56 ± 0.05% and the concentration of the amino acids in the hydrolysate ranged from 18 to 90 µg/mL.

**Conclusions:**

Previously reported biochemical methods to produce isotopically labelled proteins have been applied in the fields of proteomics and fluxomics. For these approaches, low amounts of products are required and the isotopic enrichment of the molecules has never been properly determined. So far, only ^13^C-labelled fatty acids have been isolated from labelled microalga biomass as valuable industrial products. In this study, we propose *Chlamydomonas reinhardtii* CC503 as a feasible microorganism and strain to produce labelled biomass from which a standard containing sixteen ^15^N-labelled amino acids could be obtained.

**Electronic supplementary material:**

The online version of this article (doi:10.1186/s12934-017-0759-9) contains supplementary material, which is available to authorized users.

## Background

Amino acids are a family of organic compounds containing carboxyl and amino groups. These compounds are very important not only because they are the building blocks of proteins and hence of biological structures, but also because they are involved in many metabolic pathways acting as precursors in the synthesis of porphyrins, neurotransmitters, transporting lipids, etc. [1]. Therefore, the determination of amino acids in biological samples is important not only in the food industry or drug development but also in laboratory medicine as abnormal levels of these compounds are associated with diseases or metabolic disorders [2].

Classical approaches to determine amino acids comprise high performance liquid chromatography (HPLC) coupled with pre- or post-column derivatization and fluorescence or UV/Vis detection, respectively [3]. However, there are specific applications in which high accuracy and precision are required, such as the certification of reference materials or the validation of routine analytical methods. In these cases, isotope dilution mass spectrometry (IDMS) is the preferred option as it is regarded as a primary method of measurement directly traceable to the International System of Units (SI).

The determination of amino acids by IDMS requires the availability of isotopically labelled analogues, that can be produced through a chemical [4] or a biochemical synthesis or through the hydrolysis of labelled proteins [5]. The last approach has been extensively applied to produce labelled amino acids with investigation purposes in the field of metabolomics, fluxomics and proteomics, such as the stable isotope labeling with amino acids in cell culture (SILAC) approach [6]. On the other hand, the chemical synthesis of amino acids has been more focused on the production of radioactive analogues, which find application in nuclear medicine and in anthropology [7], as only one amino acid is produced per synthetic route and the production of L-stereoisomers requires expensive chiral reagents. Amino acids can be also biotechnologically produced as metabolites, e.g. fermentation by specially developed mutants of *Corynebacterium glutamicum* or *Escherichia coli* [8]. However, only few amino acids can be obtained per synthetic route. Additionally, the microorganisms commonly used require the addition of nutrients which are a source of natural abundance isotopes leading to a decrease in the isotopic enrichment of the final products and an increase of production costs [9, 10]. Therefore, the best approach to obtain multiple labelled amino acids is to hydrolyze a previously synthesized labelled protein. This methodology has been frequently performed in metabolomic studies employing several biological species [6], however, the production of labelled amino acids with commercial purposes has not been reported thus far.

Labelled proteins have been extensively used for the structural elucidation of proteins employing multidimensional NMR [11], and hence, their production and isolation is well known. The simplest and cheapest method to obtain labelled proteins is to grow microalgae in a media containing reagents with enriched isotopes. In 2005, Fernández et al. [12] proposed a cost-effective procedure to produce ^13^C/^15^N-labelled biomass employing the algae *Phaeodactylum tricornutum*. Furthermore, they studied the suitability of the methodology to commercially produce ^13^C-labelled fatty acids from the labelled biomass. Regarding ^15^N-labelled amino acids, no studies of this type have been reported in the literature, probably because it comprises two additional complications: the isolation of proteins from the biomass and their hydrolysis.

In the present study, we report a new strategy for the biosynthesis of isotopically labelled proteins and the isolation of their constituting amino acids based on the culture of the microalgae *Chlamydomonas reindhartii* in a media containing ^15^N. The employed CC503 strain carries the nit1 and nit2 mutations so the algae cannot use nitrate or other nitrogen containing compound as a nitrogen source other than ammonium. This avoids isotopic contamination with natural abundance nitrogen isotopes. Additionally, in this strain cell walls are almost absent or produced in a negligible quantity compared to wild type. Thus, once the culture was grown, the isotopically labelled proteins could be efficiently extracted employing a specific and scaled-up version of an *in house* procedure to extract proteins from plants [13]. This work also reports the accurate determination of the isotopic enrichment of the labelled amino acids after protein hydrolysis and the quantitative results for the amino acids detected in the hydrolysate.

## Results and discussion

### Growth evaluation in a media containing ^15^N

The ease of culture and its fast growth make of *Chlamydomonas* a perfect microorganism to biosynthesize a high number of isotopically labelled compounds. *Chlamydomonas* only requires a small number of nutrients. In the case of the strain CC503 (cw92 mt+) only the nitrogen atoms from ammonium can be incorporated in their structures. For this reason, in an absence of ^14^N atoms in the initial cells the isotopic enrichment of the synthesized amino acids should be equal to that of the ^15^NH_4_Cl present in the media. As the total absence of ^14^N is not possible, we have designed a culture procedure to minimize the initial cells at the beginning of the culture. To do so, cultures where started from a single colony and were sequentially scaled up in liquid media containing ^15^NH_4_Cl.

**Fig. 1:**
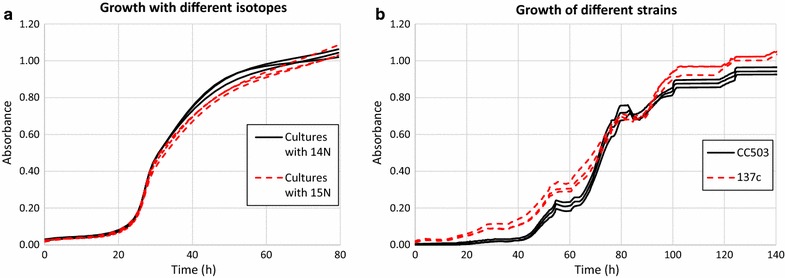
Comparison of growth curves: **a** strain CC503 in a medium with natural abundance NH_4_Cl (*solid line*) and ^15^NH_4_Cl (*dash line*), **b** strain CC503 (*solid line*) and wild type strain 137c (*dash line*) in a medium containing ^15^NH_4_Cl

Although the culture of *Chlamydomonas* in a media containing ^15^N has been extensively studied with several strains [14–16], the strain CC503 cw92 *nit1 nit2* mt+ has never been employed. Hence, it was required first to study whether kinetic isotopic effects (KIEs) were present. This was carried out by studying the growth rates in the presence of natural abundance nitrogen or enriched ^15^N. So, three cultures containing a media with natural abundance NH_4_Cl and three cultures containing enriched ^15^NH_4_Cl were grown at the same time and under the same conditions to evaluate the occurrence of isotopic effects. Growth was monitored until stationary phase by measuring the absorbance at 750 nm. The results obtained are presented in Fig. 1a. As it can be seen no differences in cell growth regarding its feeding with natural abundance N or ^15^N can be observed, demonstrating an absence of isotopic effects.

Additionally, the growth of the strain CC503 was compared with the growth of the cell-walled strain 137c (CC-125, *nit1 nit2* agg1+ mt+), both in a media containing enriched ^15^NH_4_Cl. Three cultures of each strain were grown in parallel and the growth was measured as described previously (Fig. 1b). *Chlamydomonas* strain 137c showed higher growth rates with respect to the strain CC503. However, at the end of the growth we see a time lag of approximately 24 h, where the optical density of the mutant CC503 matches with the optical density of the wild type 24 h before. In production terms, we can conclude that the use of the strain CC503 would imply larger production times to obtain the same amount of labelled biomass.

### Protein extraction

There are many approaches to extract proteins from cells, which vary depending on the sample matrix and the number, nature, and purity of the proteins to be extracted. Here, we have performed a phenol based extraction developed *in*-*house,* that has shown better results than commercial kits when extracting proteins from *Chlamydomonas* [13]. However, this procedure has been applied so far to 50 mg (fresh weight) of biomass, reporting a protein yield of 1.8 mg [13]. This amount is too low to manipulate and to detect some of the amino acids after the hydrolysis of the protein. To scale up the extraction to higher amounts of microalgae, a series of extractions starting from 900 to 1300 mg of fresh weight biomass were performed. Here again, the use of the strain CC503 was compared with the use of the wild type strain 137c. The Table 1 shows the results obtained for the extraction of five samples of the strain CC503 (A1–A5) and three samples of the strain 137c (A6–A8). For the strain CC503, an average extraction yield of 4.94% was obtained, whereas a yield of 1.90% was obtained for the wild type. The wall deficient strain CC503 shows a higher extraction efficiency as in this mutant cell walls are almost absent or produced in greatly reduced quantity compared to wild type, facilitating the extraction of proteins. Finally, in order to obtain the yield on dry weight, the water content was calculated from the lyophilization of 9 samples in a separate experiment, obtaining an average water content of 85 ± 1%. Extrapolating this value to the extracted samples, a dry weight average yield of 32.80% was obtained. This result is consistent with those reported previously for microalgae biomass profile [17].

**Table 1:** Protein extraction yields from *Chlamydomonas* achieved with the proposed procedure

Sample	Pellet mass (f. w.) (mg)	Protein mass (mg)	Extraction yield (f. w.) (%)	Extraction yield (d. w.) (%)
A1	895	39.7	4.44	28.57
A2	895	40.4	4.51	30.09
A3	895	41.0	4.58	30.54
A4	979	55.6	5.68	37.86
A5	979	52.8	5.39	35.96
A6	1.210	16.8	1.39	9.26
A7	1.215	30.5	2.51	16.74
A8	1.347	24.3	1.80	12.03

### Analysis of amino acids

**Fig. 2:**
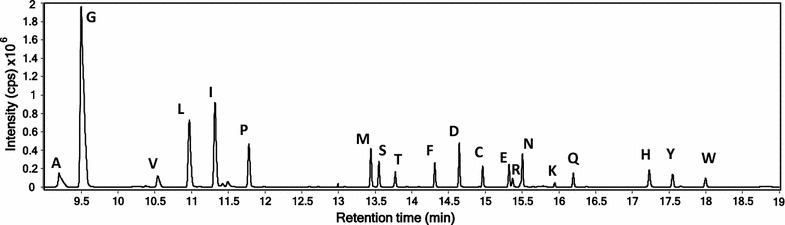
Chromatogram obtained for the separation of a mixture of amino acid standards at 10 ppm

**Fig. 3:**
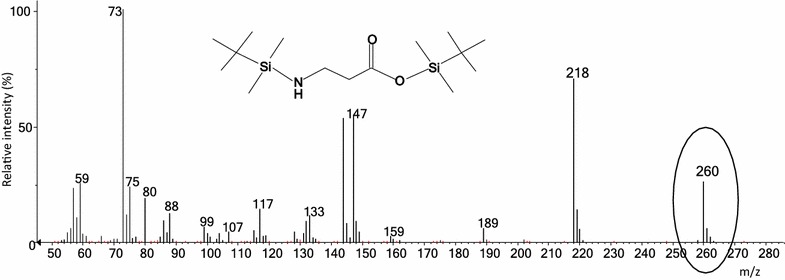
Mass spectra for alanine. The cluster selected to study the isotopic enrichment is indicated with a *circle*

GC–MS was used to determine the isotopic composition and concentration of the amino acids in the hydrolysate. For this purpose, a derivatization procedure based on the use of *N*-methyl-*N*-tert-butyldimethylsilyltrifluoroacetamide (MTBSTFA) was optimized to convert the amino acids into volatile compounds. It was found that the optimal conditions for most of the amino acids were obtained when using a ratio of reagent: acetonitrile of 1:1, 90 min of mechanical shaking and 90 °C of derivatization temperature. The identification of the derivatized amino acids was carried out first by the comparison of the experimental spectra measured in SCAN mode with Wiley and NIST MS libraries. Once the structure was proposed by the library, it was confirmed by injecting a natural abundance standard to compare the experimental mass spectra and the retention times. 20 amino acids were separated in 19 min (Fig. 2) and a pure mass cluster was selected for each of them (Fig. 3). Retention times and mass clusters employed to study the isotope enrichment are shown in Table 2. The chromatograms of the individual 20 main proteinogenic amino acid standards are presented in Additional file 1: Figure S1.

**Table 2:** Retention times and selected clusters for the 20 amino acids measured

Amino acid	Letter	r.t. (min)	Cluster
Alanine	A	9.35	258–267
Glycine	G	9.50	244–253
Valine	V	10.55	300–309
Leucine	L	10.96	300–309
Isoleucine	I	11.39	300–309
Proline	P	11.74	284–293
Methionine	M	13.45	318–327
Serine	S	13.55	388–397
Threonine	T	13.82	402–411
Phenylalanine	F	14.40	334–343
Aspartic acid	D	14.67	416–425
Cysteine	C	14.96	404–413
Glutamic acid	E	15.41	430–439
Arginine	R	15.45	182–191
Asparagine	N	15.50	415–424
Lysine	K	15.90	429–438
Glutamine	Q	16.23	429–438
Histidine	H	17.31	438–447
Tyrosine	Y	17.57	464–473
Tryptophan	W	18.03	372–381

Once the method was optimized, approximately 1 mg of the extracted proteins was hydrolyzed as described elsewhere [18] to obtain a mixture containing ^15^N-labelled amino acids. The isotopic enrichment of the labelled amino acids was determined following a previously developed procedure [19] comprising: (a) determination of the spectral purity of the selected clusters of natural abundance amino acids, (b) measurement of the isotopic composition of the labelled amino acids, (c) comparison of the measured isotope distribution of all labeled amino acids with theoretically derived distributions calculated for different tentative isotopic enrichments and, d) selection of the isotopic enrichment providing the minimum in the square sum of residuals for the linear regression between the theoretical and experimental spectra.

In the samples, 16 proteinogenic amino acids were finally identified (Fig. 3 shows the mass spectra for alanine. The mass spectras of the other 15 amino acids is presented in Additional file 2: Figure S2) and characterized in terms of isotopic enrichment and concentration. Tryptophan and cysteine were degraded during the acid digestion of the proteins and therefore could not be not detected in the hydrolysate. Asparagine and glutamine were converted to aspartic and glutamic acids during the acid digestion, respectively. The isotopic enrichments calculated for each of the detected amino acids are shown in Table 3. As can be observed, very similar isotopic enrichments were obtained for all amino acids. If we calculate the average and the associated standard deviation from all amino acids, an enrichment of 99.56 ± 0.05% was obtained which is very close to the value of 99.9% enrichment of ^15^N-ammonium chloride provided by the manufacturer. These results demonstrate that the isotopic enrichment was kept through the whole procedure, including culture, extraction, hydrolysis and analysis.

**Table 3:** Isotopic enrichment for the detected amino acids

Amino acid	Fragment formula	^15^N isotopic enrichment (%)	Concentration ± s.d. (ppm)
Alanine	C_11_H_26_^15^NO_2_Si_2_	99.68	70.50 ± 0.23
Glycine	C_10_H_24_^15^NO_2_Si_2_	99.50	45.68 ± 0.43
Valine	C_14_H_32_^15^NO_2_Si_2_	99.56	52.38 ± 0.17
Leucine	C_14_H_32_^15^NO_2_Si_2_	99.56	78.27 ± 0.12
Isoleucine	C_14_H_32_^15^NO_2_Si_2_	99.52	35.24 ± 0.10
Proline	C_13_H_28_^15^NO_2_Si_2_	99.54	44.44 ± 3.56
Methionine	C_13_H_30_^15^NO_2_SSi_2_	99.59	21.86 ± 0.02
Serine	C_17_H_40_^15^NO_3_Si_3_	99.49	34.49 ± 0.04
Threonine	C_18_H_42_^15^NO_3_Si_3_	99.54	41.06 ± 0.60
Phenylalanine	C_17_H_30_^15^NO_2_Si_2_	99.58	45.07 ± 0.12
Aspartic acid	C_18_H_40_^15^NO_4_Si_3_	99.51	77.98 ± 0.07
Glutamic acid	C_19_H_42_^15^NO_4_Si_3_	99.56	90.78 ± 0.12
Arginine	C_10_H_22_^15^NSi	99.60	57.32 ± 0.79
Lysine	C_20_H_47_^15^N_2_O_2_Si_3_	99.54	52.69 ± 1.39
Histidine	C_20_H_42_^15^N_3_O_2_Si_3_	99.56	18.48 ± 0.06
Tyrosine	C_23_H_44_^15^NO_3_Si_3_	99.64	34.14 ± 0.17

The amino acid content in the hydrolysate was assessed employing Reverse Isotope Dilution and the Certified Reference Material TraceCERT^®^ *Amino Acids Mix Solution*. This mixture contains the 16 natural abundance amino acids which are present in the hydrolysate. The concentration of each amino acid in the sample was obtained directly employing the equation of the Isotope Dilution (Eq. 1), where the molar fractions of the non-enriched amino acid (Χ*nat*) and enriched amino acid (X*enr*) were obtained through multiple linear regression from the isotopic distributions of the selected clusters for each amino acid and the concentration of the added amino acids (C*nat*) was known [19]. The hydrolysate was quantified in triplicate and each sample was measured three times. The results are shown in Table 3.

1 $$\frac{Cnat}{Cenr} = \frac{Xnat}{Xenr}$$

## Conclusions

In this study, we report an innovative, simple, and fast method to produce isotopically labelled amino acids employing *Chlamydomonas reindhartii*, which could be eventually employed as mixture of isotopically labelled amino acids in IDMS. Regarding isotope enrichment, the use of the strain CC503 cw92 mt+ and ^15^N-labelled ammonium chloride reduces the incorporation of natural abundance nitrogen, as nitrates or other nitrogen containing compounds are not incorporated as nitrogen sources. Moreover, this wall deficient mutant facilitated the extraction of proteins, leading to higher extraction yields than those obtained for a wild type *Chlamydomonas*. The average isotopic enrichment obtained for the 16 detected proteinogenic amino acids was 99.56 ± 0.05 (s.d.) % demonstrating the lack of natural abundance nitrogen incorporation through the whole procedure. The amino acid content ranged from 18–90 µg/mL, depending on the occurrence of the amino acids present in the proteins.

As the effective incorporation of ^15^N has been proved, other labelled bio-molecules should be present in the cells, and this biomass could be employed to isolate ^15^N-labelled nucleic acids and other metabolites in future experiments. Additionally, multiple labelling could be performed if ^13^C were supplied to the culture media.

## Methods

### Strains and solutions

*Chlamydomonas reinhardtii* strain CC503 cw92 *nit1 nit2* mt+ and 137c, CC125 *nit1 nit2* agg1+ mt+ were purchased from the *Chlamydomonas* Resource Center of the University of Minnesota.

All solutions were prepared using ultrapure water (bidistilled, deionized) and analytical/ultra HPLC grade reagents. TAP culture recipe consisted of 25.0 mL/L TAP salts, 2.42 g/L tris(Hidroxymethyl) aminomethane, 1 mL/L acetic acid, 0.375 mL/L phosphate solution and 1 mL/L Hutnet’s trace solution, adjusted to pH 8.0 [20]. TAP salts solution consisted of 15 g/L NH_4_Cl, 4 g/L MgSO_4_·7H_2_O and 2 g/L CaCl_2_·2H_2_O. Phosphate solution consisted of 288 g/L K_2_HPO_4_ and 144 g/L KH_2_PO_4_. Hutner’s trace solution consisted of 50 g/L EDTA, 11.40 g/L H_3_BO_3_, 22.00 g/L ZnSO_4_·7H_2_O, 5.06 g/L MnCl_2_·4H_2_O, 4.99 g/L FeSO_4_·7H_2_O, 1.61 g/L CoCl_2_·6H_2_O, 1.57 g/L CuSO_4_·5H_2_O and 1.10 g/L (NH_4_)_6_Mo_7_O_24_·4H_2_O. For isotopically enriched media, (NH_4_)_6_Mo_7_O_24_·4H_2_O was substituted for Na_6_Mo_7_O_24_·4H_2_O and NH_4_Cl was substituted for ^15^NH_4_Cl (99.9% isotope enrichment, Cambridge Isotope Laboratories, Massachusetts).

Extraction buffer prepared in situ consisted of 100 mM Tris–HCl, 10% (v/v) glycerol, 2 mM PMSF and 10 mM DTT, adjusted to pH 8.0.

Certified Reference Material TraceCERT^®^ *Amino Acids Mix Solution* was purchased from Sigma Aldrich.

### Culture procedure to maximize ^15^N isotopic enrichment

10 µL of a *Chlamydomonas* diluted solution (7500 cells/mL) containing common ^14^N-TAP media were inoculated in 390 µL of ^15^N-TAP media in a 4 mL plate. After 3 days, this culture was inoculated in 50 mL ^15^N-TAP media, and 3 days later, 1 mL of this culture was inoculated in 200 mL of ^15^N-TAP media. Additional file 3: Figure S3 shows the main steps of the whole procedure.

### Culture procedures for growth studies

To study differences in the growth of the strain CC503 in medias containing ^14^N-TAP salts and ^15^N-TAP salts, 500 µL of a *Chlamydomonas* diluted solution (7500 cells/mL) containing common ^14^N-TAP media were inoculated in 50 mL ^14^N-TAP media and after 3 days, 1 mL of this solution was inoculated in parallel to six flasks, three containing 200 mL of ^15^N-TAP media and other three containing 200 mL of ^14^N-TAP media. The cultures were performed in an HWY-200 orbital shaker (LAN Technologies, Bilbao) at 120 rpm and 25 °C, under photoperiod 16:8 light:dark and fluence rate 70 µmol/m^2^ s. Handling and inoculations were performed in a TH-100 laminar flow cabinet (Telstar, Barcelona).

To study differences in the growth of the mutant strain CC503 and the wild type strain 137c, both in medias containing ^15^N-TAP salts, 500 µL of a *Chlamydomonas* diluted solution (7500 cells/mL) containing ^14^N-TAP media were inoculated in parallel to six bioreactor flasks containing 80 mL ^15^N-TAP media. Cultures were grown in a Multicultivator MC 1000OD, PSI (Drasov, Czech Republic) with bubbling and 25 °C, under photoperiod 16:8 light:dark and rate 125 µmol/m^2^ s.

### Protein extraction

Under culture conditions described previously, by centrifuging 45 mL of culture at 4500×*g* during 3 min, pellets of around 300 mg were obtained. Three pellets were resuspended with 1 mL of water and mixed to obtain, after centrifugation, ca. 1 g (f. w.) pellets. Then, pellets were resuspended in 8 mL of extraction buffer, shaking vigorously during 1 min to homogenize. Before freezing the sample in liquid nitrogen, 400 µL of 10% SDS were added to the sample tube. Eight freeze–thaw cycles were applied. All insoluble materials were separated by centrifuging 3 min at 4500×*g* and the supernatant was transferred to a new 50 mL tube. Then, 3 mL of 2.5 M sucrose, 10 mM DTT, and 4 mL of phenol (equilibrated pH 8.0) were added. The mixture was shaken vigorously to homogenize and centrifuged 3 min at 4500×*g*. Succeeding, the upper phase (organic phase) was transferred to a new 15 mL tube. The aqueous phase was reextracted with 2 mL of phenol, repeating the previous step. The two phenol phases were mixed and cleaned by adding 2 mL of sucrose 2.5 M, 10 mM DTT. The mixture was shaken vigorously and centrifuged 3 min at 4500×*g.* The phenol phase was transferred to a new 50 mL tube with care not to disturb the interphase. The proteins were precipitated by adding 10 mL of 0.1 M ammonium acetate in methanol (5 °C) and let overnight at −20 °C. The mixture was centrifuged 3 min at 9000×*g* and the supernatant was discarded. The pellet of proteins was suspended in 5 mL of cold methanol (with 0.5% β-mercaptoethanol) and disaggregated applying ultrasounds. The mixture was centrifuged 3 min at 9000×*g* and the supernatant was discarded. This step was repeated once again with methanol and twice with acetone. Finally, the pellet of proteins was dried at 75 °C during 30 min.

### Protein hydrolysis and derivatization procedure

The hydrolysis procedure consisted of an acid hydrolysis under vacuum employing 0.5 mL of 6 N HCl [18]. Approximately 1 mg of protein was hydrolyzed during 60 h at 130 °C, employing a 6 mL vacuum hydrolysis tube (Thermo Fisher, Frankfurt) and a Heraeus furnace (Thermo Fisher).

For the derivatization, 50 µL of the hydrolysate were dried under a nitrogen stream. Then, 150 µL of ACN and 150 µL of MTBSTFA were added and taken to a ThermomixerC shaker (Eppendorf, Hamburg) at 90 °C for 90 min.

### Gas chromatography analysis of amino acids

1 µL of the derivatization mixture was injected in splitless mode in a GC (Agilent 7890A, Tokio) coupled to triple quadrupole MS (Agilent 7000). To separate the analytes a DB-5MS column (60 m × 0.25 mm × 0.25 µm) was employed. Helium was used as carrier gas with a linear velocity of 2 mL/min. The temperature program was: 120 °C (1 min), 10 °C/min to 220 °C and 20 °C/min to 300 °C (4 min).

The quantification of the amino acids was performed employing Reverse Isotope Dilution. For this purpose, a certified solution containing 17 natural abundance amino acids was used to prepare a solution in a concentration range of 19–44 ppm. Then, a mixture of 50 µL of this solution and 50 µL of the hydrolysate was prepared. This mixture was treated and injected in the GC–MS as described for the hydrolysate samples.

## Additional files

**Additional file 1: Figure S1.** Chromatograms of the individual 20 main proteinogenic amino acid standards at 50 ppm.

**Additional file 2: Figure S2.** Mass spectra obtained for 15 amino acids identified in the hydrolysate.

**Additional file 3: Figure S3.** Scheme picture of the main steps for the proposed approach.

## Abbreviations

KIE: kinetic isotope effect
HPLC: high performance liquid chromatography
GC: gas chromatography
SILAC: stable isotope labelling by amino acids in cell culture
DTT: dithiothreitol
PMSF: phenylmethylsulfonyl fluoride
MTBSTFA: *N*-methyl-*N*-tert-butyldimethylsilyltrifluoroacetamide
ACN: acetonitrile
SRMs: standard reference materials
IDMS: isotope dilution mass spectrometry
