# Supplementary material for: A cost-effective approach to produce 15N-labelled amino acids employing Chlamydomonas reinhardtii CC503
Source: Microb Cell Fact. 2017 Aug 18;16:146. doi: 10.1186/s12934-017-0759-9 (PMC5563056; doi:10.1186/s12934-017-0759-9)
Supplement: Supplementary file 1 — Additional file 1: Figure S1. Chromatograms of the individual 20 main proteinogenic amino acid standards at 50 ppm. [file 12934_2017_759_MOESM1_ESM.docx]

Figure S1. Individual chromatograms of the 20 most abundant proteinogenic amino acids, obtained employing standards at 50 ppm.
