# Supplementary material for: A cost-effective approach to produce 15N-labelled amino acids employing Chlamydomonas reinhardtii CC503
Source: Microb Cell Fact. 2017 Aug 18;16:146. doi: 10.1186/s12934-017-0759-9 (PMC5563056; doi:10.1186/s12934-017-0759-9)
Supplement: Supplementary file 2 — Additional file 2: Figure S2. Mass spectra obtained for 15 amino acids identified in the hydrolysate. [file 12934_2017_759_MOESM2_ESM.pptx]

## Slide 1
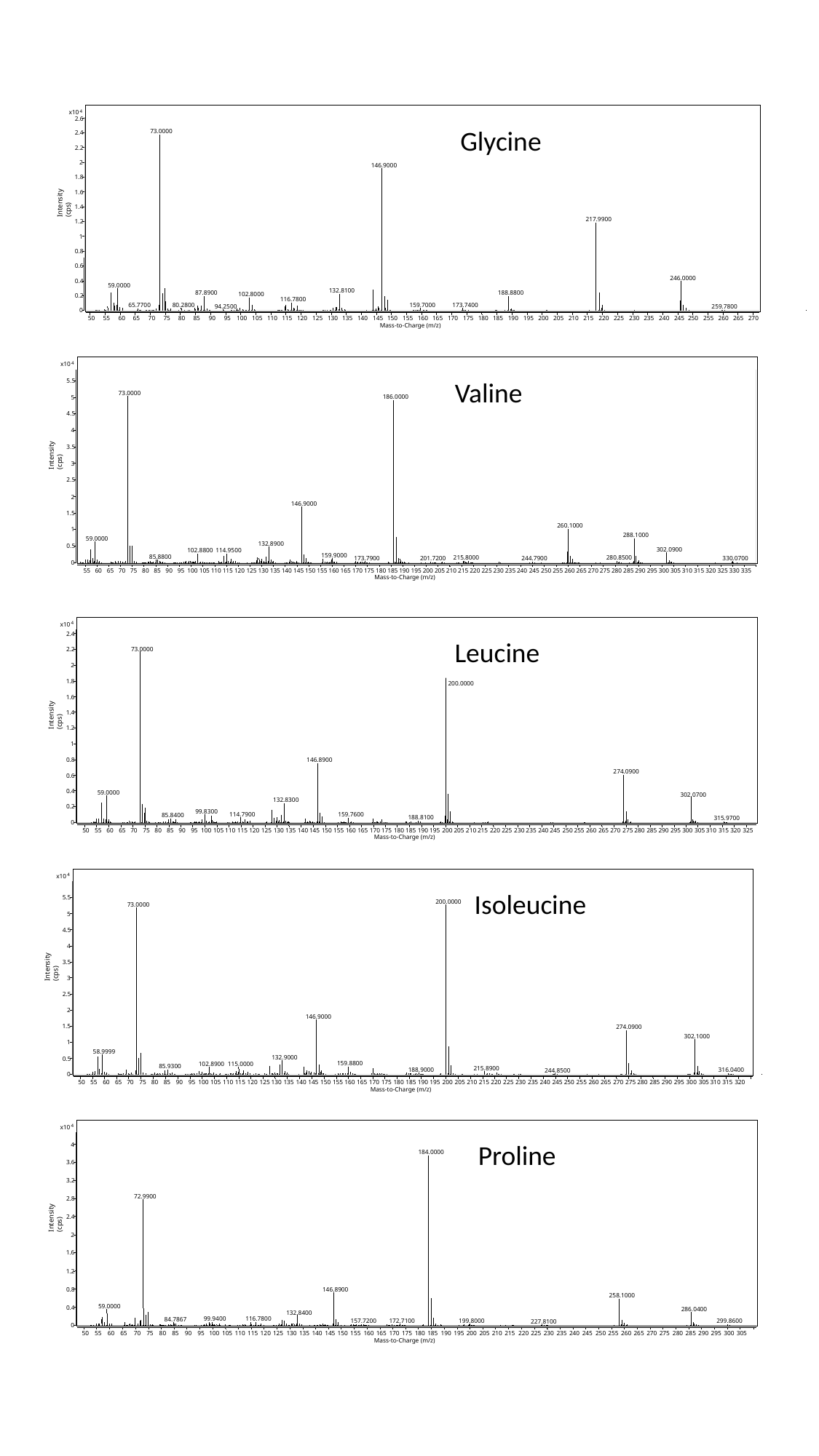

4
x10
2.6
2.4
2.2
2
1.8
1.6
1.4
1.2
1
0.8
0.6
0.4
0.2
0
73.0000
146.9000
217.9900
246.0000
59.0000
132.8100
87.8900
188.8800
102.8000
116.7800
65.7700
80.2800
159.7000
173.7400
94.2500
259.7800
50
55
60
65
70
75
80
85
90
95
100
105
110
115
120
125
130
135
140
145
150
155
160
165
170
175
180
185
190
195
200
205
210
215
220
225
230
235
240
245
Mass-to-Charge (m/z)
Glycine
250
255
260
265
270
Intensity (cps)
4
x10
5.5
5
4.5
4
3.5
3
2.5
2
1.5
1
0.5
0
Valine
73.0000
186.0000
Intensity (cps)
146.9000
260.1000
288.1000
59.0000
132.8900
302.0900
102.8800
114.9500
159.9000
85.8800
215.8000
280.8500
173.7900
201.7200
244.7900
330.0700
55
60
65
70
75
80
85
90
95
100
105
110
115
120
125
130
135
140
145
150
155
160
165
170
175
180
185
190
195
200
205
210
215
220
225
230
235
240
245
250
255
260
265
270
275
280
285
290
295
300
305
310
315
320
325
330
335
Mass-to-Charge (m/z)
73.0000
200.0000
146.8900
274.0900
59.0000
302.0700
132.8300
99.8300
114.7900
159.7600
85.8400
188.8100
315.9700
50
55
60
65
70
75
80
85
90
Mass-to-Charge (m/z)
4
x10
2.4
Leucine
2.2
2
Intensity (cps)
1.8
1.6
1.4
1.2
1
0.8
0.6
0.4
0.2
0
95
100
105
110
115
120
125
130
135
140
145
150
155
160
165
170
175
180
185
190
195
200
205
210
215
220
225
230
235
240
245
250
255
260
265
270
275
280
285
290
295
300
305
310
315
320
325
4
x10
Isoleucine
5.5
200.0000
73.0000
5
4.5
Intensity (cps)
4
3.5
3
2.5
2
146.9000
1.5
274.0900
302.1000
1
58.9999
132.9000
0.5
159.8800
102.8900
115.0000
85.9300
215.8900
188.9000
316.0400
244.8500
0
50
55
60
65
70
75
80
85
90
95
100
105
110
115
120
125
130
135
140
145
150
155
160
165
170
175
180
185
190
195
200
205
210
215
220
225
230
235
240
245
250
255
260
265
270
275
280
285
290
295
300
305
310
315
320
Mass-to-Charge (m/z)
4
x10
Proline
4
184.0000
3.6
3.2
Intensity (cps)
72.9900
2.8
2.4
2
1.6
1.2
0.8
146.8900
258.1000
59.0000
0.4
286.0400
132.8400
99.9400
116.7800
84.7867
172.7100
199.8000
157.7200
299.8600
227.8100
0
50
55
60
65
70
75
80
85
90
95
100
105
110
115
120
125
130
135
140
145
150
155
160
165
170
175
180
185
190
195
200
205
210
215
220
225
230
235
240
245
250
255
260
265
270
275
280
285
290
295
300
305
Mass-to-Charge (m/z)

## Slide 2
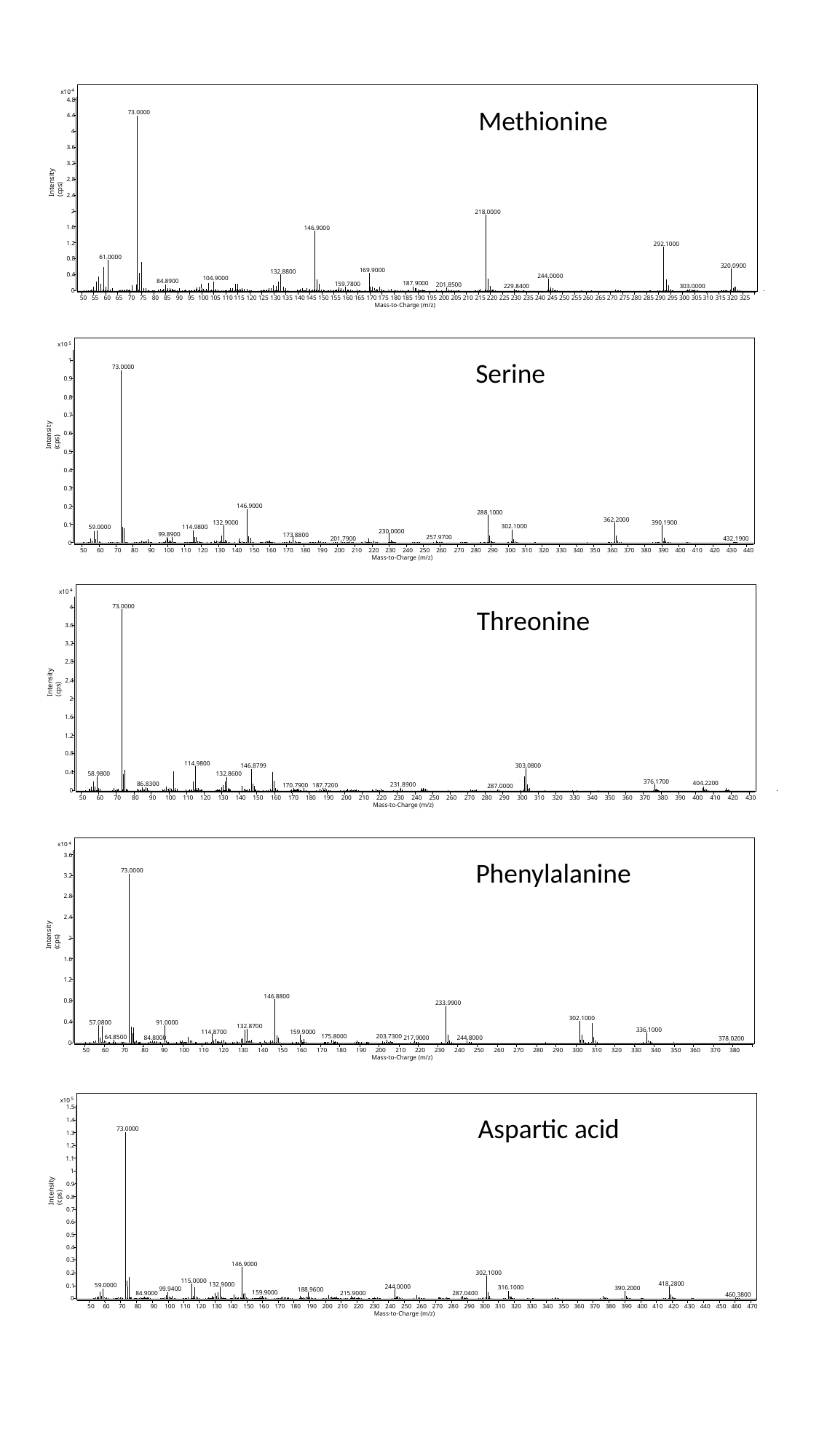

4
x10
4.8
Methionine
73.0000
4.4
4
3.6
Intensity (cps)
3.2
2.8
2.4
2
218.0000
1.6
146.9000
1.2
292.1000
61.0000
0.8
320.0900
169.9000
132.8800
0.4
244.0000
104.9000
84.8900
187.9000
159.7800
201.8500
229.8400
303.0000
0
50
55
60
65
70
75
80
85
90
95
100
105
110
115
120
125
130
135
140
145
150
155
160
165
170
175
180
185
190
195
200
205
210
215
220
225
230
235
240
245
250
255
260
265
270
275
280
285
290
295
300
305
310
315
320
325
Mass-to-Charge (m/z)
5
x10
Serine
1
73.0000
0.9
0.8
Intensity (cps)
0.7
0.6
0.5
0.4
0.3
146.9000
0.2
288.1000
362.2000
132.9000
390.1900
0.1
302.1000
59.0000
114.9800
230.0000
99.8900
173.8800
257.9700
201.7900
432.1900
0
50
60
70
80
90
100
110
120
130
140
150
160
170
180
190
200
210
220
230
240
250
260
270
280
290
300
310
320
330
340
350
360
370
380
390
400
410
420
430
440
Mass-to-Charge (m/z)
4
x10
Threonine
73.0000
4
3.6
3.2
Intensity (cps)
2.8
2.4
2
1.6
1.2
0.8
114.9800
146.8799
303.0800
0.4
58.9800
132.8600
376.1700
404.2200
86.8300
231.8900
170.7900
187.7200
287.0000
0
50
60
70
80
90
100
110
120
130
140
150
160
170
180
190
200
210
220
230
240
250
260
270
280
290
300
310
320
330
340
350
360
370
380
390
400
410
420
430
Mass-to-Charge (m/z)
4
x10
Phenylalanine
3.6
73.0000
3.2
2.8
Intensity (cps)
2.4
2
1.6
1.2
146.8800
0.8
233.9900
302.1000
0.4
57.0800
91.0000
132.8700
336.1000
114.8700
159.9000
175.8000
203.7300
64.8500
84.8000
217.9000
244.8000
378.0200
0
50
60
70
80
90
100
110
120
130
140
150
160
170
180
190
200
210
220
230
240
250
260
270
280
290
300
310
320
330
340
350
360
370
380
Mass-to-Charge (m/z)
5
x10
1.5
Aspartic acid
1.4
73.0000
1.3
1.2
Intensity (cps)
1.1
1
0.9
0.8
0.7
0.6
0.5
0.4
0.3
146.9000
302.1000
0.2
115.0000
418.2800
132.9000
59.0000
0.1
244.0000
316.1000
390.2000
99.9400
188.9600
159.9000
84.9000
215.9000
287.0400
460.3800
0
50
60
70
80
90
100
110
120
130
140
150
160
170
180
190
200
210
220
230
240
250
260
270
280
290
300
310
320
330
340
350
360
370
380
390
400
410
420
430
440
450
460
470
Mass-to-Charge (m/z)

## Slide 3
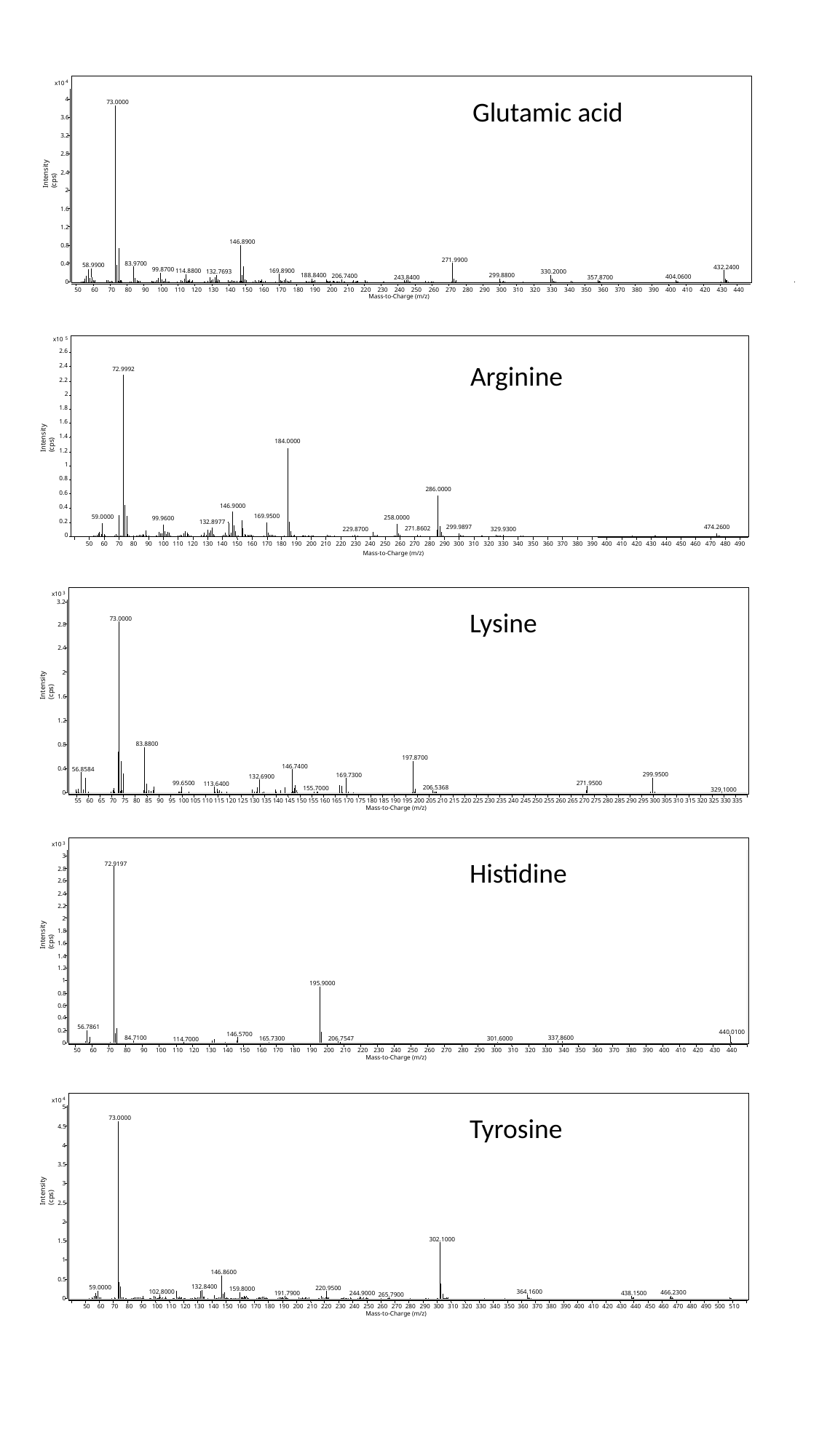

4
x10
Glutamic acid
4
73.0000
3.6
3.2
Intensity (cps)
2.8
2.4
2
1.6
1.2
146.8900
0.8
271.9900
0.4
83.9700
58.9900
432.2400
99.8700
114.8800
169.8900
132.7693
330.2000
188.8400
299.8800
206.7400
404.0600
243.8400
357.8700
0
50
60
70
80
90
100
110
120
130
140
150
160
170
180
190
200
210
220
230
240
250
260
270
280
290
300
310
320
330
340
350
360
370
380
390
400
410
420
430
440
Mass-to-Charge (m/z)
x10
5
2.6
Arginine
2.4
72.9992
2.2
2
Intensity (cps)
1.8
1.6
1.4
184.0000
1.2
1
0.8
286.0000
0.6
146.9000
0.4
169.9500
59.0000
258.0000
99.9600
0.2
132.8977
299.9897
474.2600
271.8602
229.8700
329.9300
0
50
60
70
80
90
100
110
120
130
140
150
160
170
180
190
200
210
220
230
240
250
260
270
280
290
300
310
320
330
340
350
360
370
380
390
400
410
420
430
440
450
460
470
480
490
Mass-to-Charge (m/z)
3
x10
3.2
Lysine
73.0000
2.8
2.4
Intensity (cps)
2
1.6
1.2
83.8800
0.8
197.8700
146.7400
0.4
56.8584
299.9500
169.7300
132.6900
99.6500
271.9500
113.6400
206.5368
155.7000
329.1000
0
55
60
65
70
75
80
85
90
95
100
105
110
115
120
125
130
135
140
145
150
155
160
165
170
175
180
185
190
195
200
205
210
215
220
225
230
235
240
245
250
255
260
265
270
275
280
285
290
295
300
305
310
315
320
325
330
335
Mass-to-Charge (m/z)
3
x10
Histidine
3
72.9197
2.8
2.6
2.4
Intensity (cps)
2.2
2
1.8
1.6
1.4
1.2
1
195.9000
0.8
0.6
0.4
56.7861
0.2
440.0100
146.5700
84.7100
337.8600
206.7547
165.7300
301.6000
114.7000
0
50
60
70
80
90
100
110
120
130
140
150
160
170
180
190
200
210
220
230
240
250
260
270
280
290
300
310
320
330
340
350
360
370
380
390
400
410
420
430
440
Mass-to-Charge (m/z)
4
x10
5
Tyrosine
73.0000
4.5
4
Intensity (cps)
3.5
3
2.5
2
302.1000
1.5
1
146.8600
0.5
132.8400
59.0000
220.9500
159.8000
102.8000
364.1600
466.2300
191.7900
244.9000
438.1500
265.7900
0
50
60
70
80
90
100
110
120
130
140
150
160
170
180
190
200
210
220
230
240
250
260
270
280
290
300
310
320
330
340
350
360
370
380
390
400
410
420
430
440
450
460
470
480
490
500
510
Mass-to-Charge (m/z)
