# Supplementary material for: A cost-effective approach to produce 15N-labelled amino acids employing Chlamydomonas reinhardtii CC503
Source: Microb Cell Fact. 2017 Aug 18;16:146. doi: 10.1186/s12934-017-0759-9 (PMC5563056; doi:10.1186/s12934-017-0759-9)
Supplement: Supplementary file 3 — Additional file 3: Figure S3. Scheme picture of the main steps for the proposed approach. [file 12934_2017_759_MOESM3_ESM.pptx]

## Slide 1
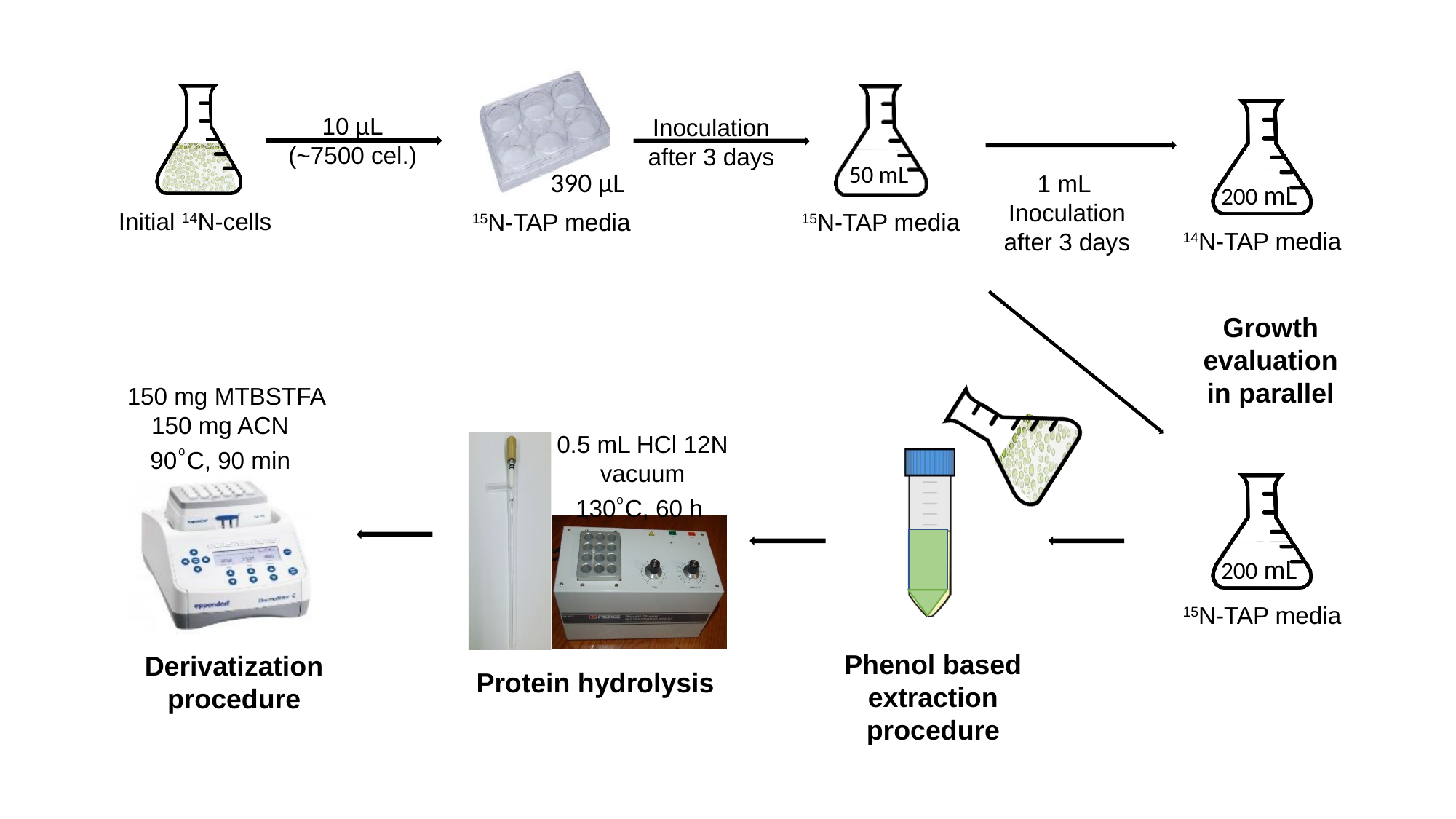

390 µL
50 mL
200 mL
10 µL
(~7500 cel.)
Inoculation
after 3 days
1 mL
Inoculation
after 3 days
Initial 14N-cells
15N-TAP media
15N-TAP media
14N-TAP media
Growth evaluation in parallel
150 mg MTBSTFA
150 mg ACN
0.5 mL HCl 12N
vacuum
130 ͦ C, 60 h
90 ͦ C, 90 min
200 mL
15N-TAP media
Phenol based extraction procedure
Derivatization procedure
Protein hydrolysis
